# Cooperative targeting of PARP-1 domains to regulate metabolic and developmental genes

**DOI:** 10.3389/fendo.2023.1152570

**Published:** 2023-06-06

**Authors:** Gbolahan Bamgbose, Sarah Johnson, Alexei Tulin

**Affiliations:** Department of Biomedical Sciences, School of Medicine and Health Sciences, University of North Dakota, Grand Forks, ND, , United States

**Keywords:** PARP-1, metabolic genes, developmental genes, gene expression, PARP-1 domains

## Abstract

PARP-1, also known as poly(ADP-ribose) polymerase 1, is a multifunctional nuclear enzyme that plays a critical role in transcriptional regulation through its three functional domains: the N-terminal DNA-binding domain (DBD) containing two zinc fingers for DNA binding and a third zinc finger for maintaining interdomain contacts, the auto modification domain (AD), and the C-terminal domain, which includes the protein-interacting WGR domain and the catalytic domain. Despite the critical role that PARP-1 plays in regulating gene expression, the mechanisms by which it is targeted to chromatin are not well understood. In this study, we aimed to understand the targeting of PARP-1 to chromatin using ChIP-seq of YFP-tagged deletional isoforms of PARP-1 (ZnI, ZnII, AD-WGR) and a construct that lacks only ZnI (ΔZnI). Our results indicate that other PARP-1 domains are sufficient to target PARP-1 to active genes in the absence of ZnI. Furthermore, we found that PARP-1 represses metabolic gene pathways and activates developmental gene pathways. The results of ChIP-seq analysis showed that PARP-1 and ΔZnI were preferentially bound to the gene bodies of PARP-1-regulated metabolic genes compared to developmental genes. PARP-1 domains (ZnI, ZnII and AD-WGR) also preferentially occupied the gene bodies of PARP-1-regulated metabolic genes, however, they were more enriched at the TSS of PARP-1-regulated developmental genes compared to metabolic genes. Thus, we propose that PARP-1 domains cooperatively target PARP-1 to PARP-1-regulated genes to coordinate metabolic and developmental gene expression programs.

## Introduction

PARP-1 is a well-known poly (ADP-ribose) polymerase that plays a crucial role in various cellular processes, including DNA repair (1–3), transcriptional regulation (4–6) and a host of biological processes. It is also one of the most abundant proteins found in the eukaryotic nucleus, second only to histones (1). PARP-1 is a member of the PARP family of enzymes, which are known for their ability to add poly(ADP-ribose) (PAR) to target proteins (1–3). When PARP-1 is activated by DNA damage, developmental or environmental cues, it attaches a negatively charged polymeric chain of ADP-ribose units predominantly onto itself and to a variety of proteins, including chromatin-associated proteins to modulate their function or remodel chromatin (5–8).

PARP-1 is composed of three main functional domains: the N-terminal DNA-binding domain (DBD), the automodification domain (AD), and the C-terminal domain (1, 9). The N-terminal DBD contains two zinc fingers that are responsible for binding to DNA and a third zinc finger that maintains interdomain contacts (1, 10). The automodification domain is automodified with PAR by PARP-1, while the C-terminal domain includes the WGR domain, which regulates protein-protein interactions, and the catalytic domain (CAT), which is involved in the PARylation of chromatin-associated proteins and other target proteins (1, 9). The mechanisms by which these domains target PARP-1 to chromatin to facilitate transcriptional regulation is not completely understood.

In this study, we employed genome-wide methods to investigate how PARP-1 is directed to chromatin by PARP-1 domains in the third-instar larvae of *Drosophila*. We demonstrated that the ZnI domain of PARP-1 is not necessary for targeting to chromatin, and other PARP-1 domains are sufficient for targeting PARP-1 to chromatin. Furthermore, we showed that at differentially expressed genes in *Parp^C03256^* mutants, PARP-1 preferentially binds to the gene bodies of upregulated metabolic genes, while the ZnI, ZnII, and AD-WGR domains preferentially target PARP-1 to the gene bodies of upregulated metabolic genes and the TSS of downregulated developmental genes lacking H2Av enrichment. These results demonstrate that PARP-1 domains cooperatively target genes regulated by PARP-1.

## Materials and methods

### Fly husbandry and genetics

Flies were cultured on standard cornmeal-molasses agar in a 25°C incubator. All fly stocks were obtained from Bloomington Drosophila Stock Center and the Exelixis collection at the Harvard Medical School unless otherwise stated. Wandering third-instar larvae were used for all experiments. The *Parp* ^C03256^ was generated in a single pBac-element mutagenesis screen (11). UAS::PARP-1-EYFP, UAS::ΔZnI-EYFP, UAS::ZnI-EYFP, UAS::ZnII-EYFP, and UAS::AD-WGR-EYP constructs were previously described (12) and were expressed using 69B-GAL4 driver (13). W^1118^ strains were used as controls for ChIP-seq and RNA-seq and termed WT as appropriate. We used the tubby balancer to isolate *Parp* ^C03256^ homozygous mutants.

### Chromatin immunoprecipitation sequencing

75 wandering third-instar larvae (3 biological replicates per genotype) were collected in a 2ml DNA LoBind Eppendorf tube and washed twice with 1ml 1X PBS. The larvae were homogenized in ice-cold lysis buffer (200ul 1X protease inhibitor cocktail, 250ul PMSF, 800ul 1X PBS, 1ul Tween 20) using a pellet pestle. The homogenized lysate was supplemented with 244.5ul of 11% formaldehyde to a final concentration of 1.8% and the samples were crosslinked for 15 minutes at room temperature on a rotator. Glycine was added to a final concentration of 500mM to quench the fixative on ice for 5 minutes at room temperature. The larval debris was pelleted at 1,000g for 3 minutes and the supernatant was removed. The pellet was resuspended in 1ml sonication buffer (0.5% SDS, 20mM Tris pH 8.0, 2mM EDTA, 0.5mM EGTA, 0.5mM PMSF, 1X protease inhibitor cocktail) and chromatin was fragmented to 300 – 500bp Bioruptor sonicator (UCD-200) for 20 cycles (30 seconds high frequency sonication, 1.5 sec pause) in a cold room. The sonicated material was pelleted at 10,000g for 10 minutes at 4°C, supernatant was collected, then fragment size was checked prior to immunoprecipitation. The sonicated chromatin was pre-cleared and incubated with anti-GFP antibody overnight (TP-401, Torrey Pines Biolabs) 4°C overnight. The immunoprecipitated chromatin was then collected with pre-washed Protein A agarose beads for 2 hours. The beads were sequentially washed with the following buffers: 1 low salt buffer wash (0.1% SDS, 1% Triton X-100, 2mM EDTA, 20mM Tris-HCL pH 8.0, 150mM NaCl), 3 high salt buffer washes (0.1% SDS, 1% Triton X-100, 2mM EDTA, 20mM Tris-HCL pH 8.0, 500mM NaCl), 1 LiCL wash (2mM EDTA, 20mM Tris-HCl pH 8.0, 0.25M LiCl, 1% NP-40) and 2 TE buffer washers before elution. Bound chromatin on beads was eluted twice at room temperature using 250ul of freshly prepared ChIP elution buffer (1% SDS, 100mM NaHCO3) for 15 minutes and reverse-crosslinked overnight. The eluates were then treated with RNase A and proteinase K prior to DNA extraction *via* phenol-chloroform extraction and ethanol precipitation. Libraries were made and sequenced at Novogene.

### ChIP-seq analysis

The quality of FASTQ files (raw reads) were checked using FastQC (version. 0.11.9) and adapters were removed with fastp (14). Trimmed FASTQ files were aligned to the *Drosophila* genome (dm6) using Bowtie2 to generate bam files (15). Unmapped and low-quality reads were discarded from bam files (<=20 mapQuality) using BamTools (16). Duplicate reads were identified and removed from mapped reads using Picard MarkDuplicates (http://broadinstitute.github.io/picard/). Deeptools MultiBamSummary was used to determine reproducibility of ChIP-Seq reads. MACS2 was used to call peaks against pooled Input/control using default settings except narrowPeaks were called for PARP-1, ZnI, ZnII, AD-WGR, Pol II, Pol II Ser2p, H3K9ac, H3K27ac and broadPeaks were called for H2Av, H3K9me2 and H3K9me3. MACS2 bedGraph pileups were used to generate normalized coverage of ChIP-seq signals using Deeptools bigWigCompare by computing the ratio of the signals (IP vs Control/Input) using a 50bp bin size. Deeptools plotHeatmap was used to create enrichment profiles across promoters ( ± 2kb) reference point mode (TSS) using a 50bp bin size. Genic enrichment profiles were determined using scaled region mode. PARP-1 clusters were determined using K means function (n=4) in Deeptools computematrix (17). Homer suite was used for *de novo* motif analysis (18) at promoters (TSS ± 500) using the findMotifsGenome.pl function and the following parameters -mask -size given -S 10.

### RNA-seq

RNA was isolated from 10 wandering third-instar larvae (3 biological replicates per genotype; WT and *Parp^C03256^*) using RNeasy lipid tissue mini kit (Qiagen). RNA samples were flash-frozen in liquid nitrogen and sent to Novogene for library preparation and sequencing. mRNA was purified from total RNA *via* poly-T oligo beads. Libraries were prepared using Ultra II RNA library kit (NEB) and samples were sequenced on the NovaSeq 6000 platform (Illumina) at Novogene.

### RNA-seq analysis

Paired-end reads were quality-checked using FastQC and reads were mapped to the *Drosophila* genome (dm6) using RNA STAR (19). Reads per annotated gene was counted using featureCounts (20). Differential expression analysis was performed with DESeq2 (21), with Log_2_ fold change of at least 1 (absolute) considered significant (FDR < 0.05). Volcano plots of top 20 differentially expressed genes was generated with ggplot2 (version 3.3.3). To visualize RNA-seq data, bam files were converted to bigwig with deeptools bamCoverage using default parameters and normalizing to reads per kilobase per million (RPKM).

### Chip-seq and RNA-seq visualization

IGV (2.13.1) was used to visualize bigwig files of ChIP-seq and RNA-seq.

### Gene ontology and Gene set enrichment analysis

Gene ontology terms were determined using g:profiler (FDR<0.05) (22). GSEA analysis was performed against gene sets determined in this study or gene sets imported from https://biit.cs.ut.ee/gprofiler/static/gprofiler_dmelanogaster.name.zip using GSEA software (version.4.2.2) (23, 24).

## Results

### ZnI is dispensable for PARP-1 targeting to chromatin at steady state

**Figure 1:**
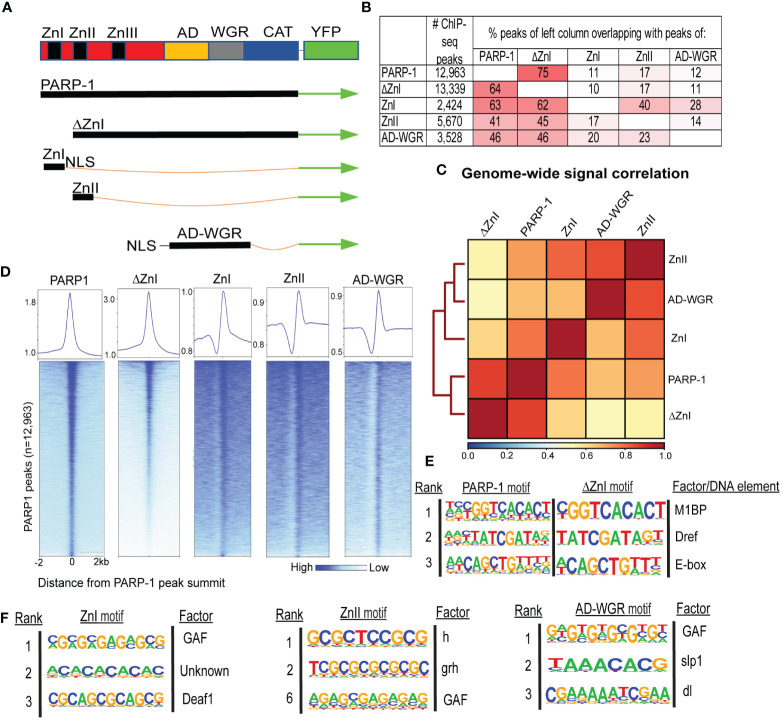
ZnI domain is not required to target PARP-1 to chromatin at steady state. **(A)** Schema showing YFP-tagged PARP-1, ΔZnI, and deletional isoforms. **(B)** Fraction of overlap of annotated peaks. Percentages greater than 10% are highlighted in red. **(C)** Spearman correlation of genome-wide normalized ChIP-seq signals of PARP-1, ΔZnI and PARP-1 domains. The key shows the spearman correlation coefficients. **(D)** Heatmap showing colocalization of PARP-1, ΔZnI and PARP-1 domains at PARP-1 peaks (n = 12,963). The upper plots show the summary of the heatmap signals. **(E)** HOMER motif analysis of PARP-1 and ΔZnI peaks. Top 3 significant promoter motifs and their associated factors/DNA element are shown. **(F)** HOMER motif analysis of PARP-1 domain (ZnI, ZnII and AD-WGR) peaks. Top significant motifs and their associated factors are shown.

We performed ChIP-seq using PARP-1, ΔZnI and PARP-1 domains (ZnI, ZnII, AD-WGR) fused with YFP in *Drosophila* third-instar larvae to determine how PARP-1 is targeted to chromatin (**Figure 1A**). To our surprise, there was high overlap between PARP-1 and ΔZnI (75%-64%) peaks (**Figure 1B**). ZnI, ZnII and AD-WGR domain peaks highly overlapped PARP-1 peaks (63%-41%-46%) and ΔZnI peaks (62%-45%-46%) at a similar frequency (**Figure 1B**). We observed a high correlation between PARP-1, ΔZnI and PARP-1 domains ChIP-seq signals genome-wide (**Figure 1C**). Consistently, ΔZnI and PARP-1 domains were highly colocalized with PARP-1 ChIP-seq signals at PARP-1 peaks (**Figure 1D**). Furthermore, motif analysis showed that PARP-1 and ΔZnI bind the same motifs, particularly motif 1 which is associated with Motif 1 binding protein (M1BP) (25), DNA replication-related element factor (Dref) motif (26) and the enhancer box DNA element (E-box) (**Figure 1E**). This finding implies that the ZnI domain is not essential for steady-state PARP-1 targeting to chromatin. PARP-1 domains peaks were associated with motifs of transcription factors associated with developmental processes; these factors include Deformed epidermal autoregulatory factor-1 (Deaf1) (27), Hairless (h) (28), Grainyhead (grh) (29), sloppy paired 1 (slp1) (30), dorsal (dl) (31), and notably, the GAGA factor (GAF) (32) (**Figure 1F**). Thus, PARP-1 domains may target PARP-1 to the promoters of developmental genes.

**Figure 2:**
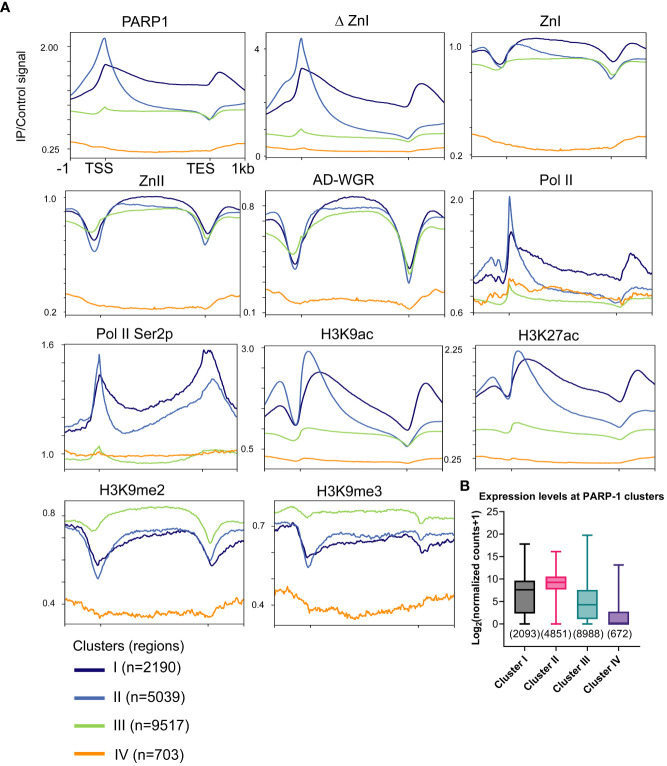
ZnI is dispensable for PARP-1 targeting to the promoters of active genes. **(A)** Metagene profiling showing normalized ChIP-seq signals of PARP-1, ΔZnI, PARP-1 domains, Pol II, Pol II Ser2p and histone modifications at PARP-1 gene clusters. **(B)** Boxplot showing expression levels of genes in PARP-1 clusters. Box plot: dashed center line, median; box-plot limits, upper and lower quartiles; whiskers, minimum and maximum values.

Next, we examined the distribution of PARP-1 in a gene-centric manner using k-means clustering. PARP-1 was highly enriched at the promoters of genes in clusters I and II (**Figure 2A**). Genes in clusters I and II were highly enriched with active chromatin signatures such as Pol II, elongating Pol II Ser2p, H3K9ac and H3K27ac (**Figure 2A**). Likewise, ΔZnI was highly enriched at active gene clusters (I and II) and had a similar binding profile to PARP-1 (**Figure 2A**). PARP-1 domains were specifically enriched at the gene bodies of genes in clusters I and II, suggesting they may target PARP-1 to the gene bodies of active genes (**Figure 2A**). However, PARP-1 domains were depleted from the TSS of active gene clusters, suggesting the CAT, ZnIII domains, or a combination of all domains besides ZnI may be required for PARP-1 targeting to active promoters (**Figure 2A**).

PARP-1 domains were more enriched at cluster III compared to PARP-1 and ΔZnI (**Figure 2A**). Cluster III genes were highly enriched with H3K9me2 and H3K9me3 repressive histone marks compared to active chromatin signatures like H3K9ac and H3K27ac suggesting that cluster III genes are expressed at lower levels (**Figure 2A**). Cluster IV genes are unmarked suggesting they are low expression genes. Consistently, cluster I and II genes were highly expressed while cluster III and IV genes were lowly expressed (**Figure 2B**). Thus, even though ZnI, ZnII and AD-WGR preferentially target PARP-1 to gene bodies of active genes, they may also target PARP-1 to low expression genes at steady state. Overall, our data suggests that ZnI is not required for PARP-1 targeting to chromatin at steady state and other PARP-1 domains are sufficient to fulfill this role.

### PARP-1 domains cooperatively target genes regulated by PARP-1

**Figure 3:**
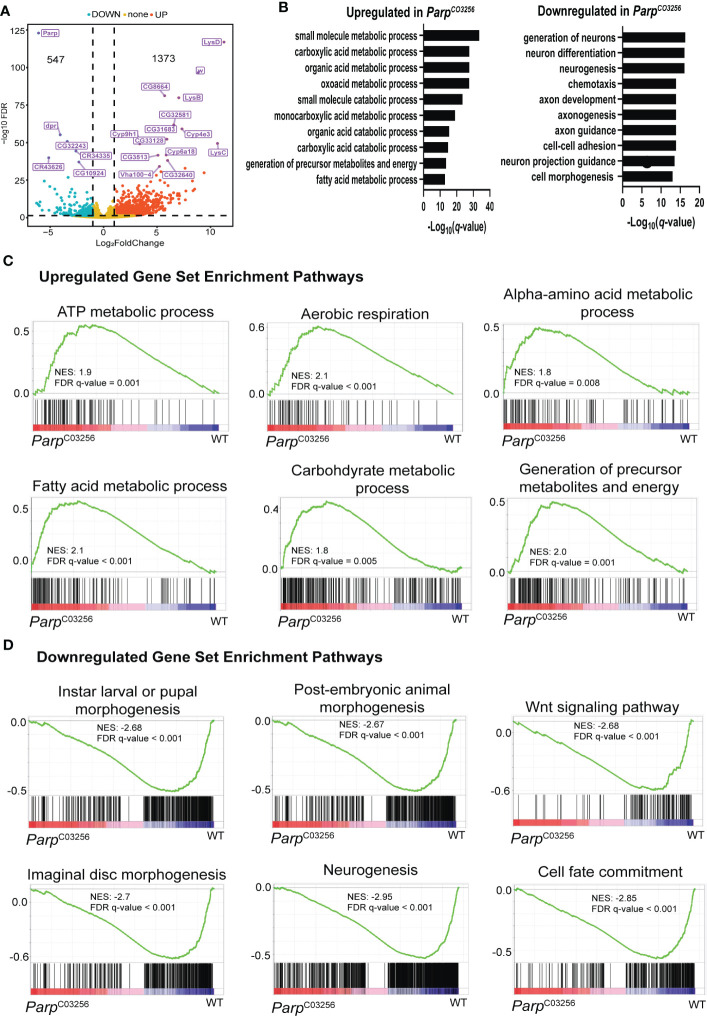
PARP-1 represses metabolic gene pathways and activates developmental gene pathways **(A)** Volcano plot showing the top 20 differentially expressed genes in *Parp^C03256^*. **(B)** Gene ontology of differentially DEGs in *Parp^C03256^ mutant* animals. GSEA analysis showing pathways that were **(C)** upregulated and **(D)** downregulated in *Parp^C03256^* mutant animals.

To understand how PARP-1 domains target PARP-1 to genes regulated by PARP-1, we examined the binding pattern of PARP-1 domains at differentially expressed genes (DEGs) in *Parp* ^C03256.^third-instar larvae. This hypomorph mutant strain exhibits significantly low expression of total PARP-1 protein lacking its first zinc finger (10). Additionally, this strain undergoes developmental arrest during larval-to-pupal transition. Gene ontology analysis of DEGs in *Parp^C03256^* mutants showed that metabolic genes were upregulated while developmental genes, particularly genes involved in neural development and differentiation, were downregulated. (**Figures 3A, B**). Gene set enrichment analysis was consistent with the PARP-1 regulated transcriptome. Pathways for metabolic processes were upregulated in *Parp^C03256^* mutant animals (**Figure 3C**) while pathways for larval morphogenesis and neurogenesis were downregulated (**Figure 3D**).

**Figure 4:**
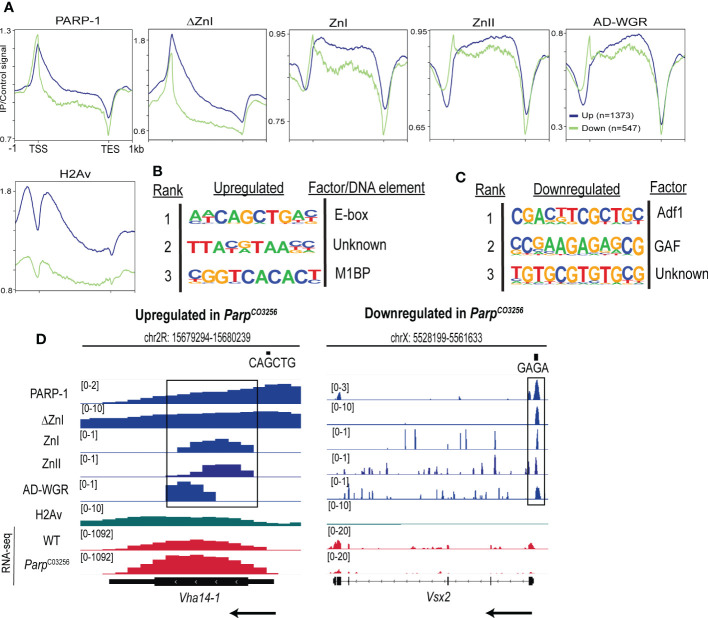
PARP-1 domains cooperate to target PARP-1 to PARP-1-regulated metabolic and developmental genes. **(A)** Metagene plots showing genic enrichment of PARP-1, ΔZnI, PARP-1 domains and H2Av ChIP-seq signal at upregulated (n=1373) and downregulated (n=547) genes in *Parp^C03256^*. HOMER motif analysis of **(B)** upregulated and **(C)** downregulated genes in *Parp^C03256^* that were highly occupied by PARP-1 **(D)** Representative IGV tracks of PARP-1, ΔZnI, PARP-1 domains and H2Av at differentially expressed genes in *Parp^C03256^.* Black rectangles show regions of colocalization. Black arrows indicate direction of transcription.

Next, we determined the enrichment of PARP-1 and PARP-1 domains at PARP-1-regulated genes. PARP-1 preferentially binds the gene bodies of upregulated genes compared to downregulated genes (**Figure 4A**). Likewise, ΔZnI had a similar binding profile to PARP-1 at PARP-1-regulated genes (**Figure 4A**). Consistently, PARP-1 domains preferentially occupied the gene bodies of upregulated genes compared to downregulated genes (**Figure 4A**). However, PARP-1 domains had a punctate enrichment at the TSS of downregulated genes (**Figure 4A**). Interestingly, the histone H2A variant, H2Av, was significantly diminished in downregulated genes compared to upregulated genes (**Figure 4A**). We previously showed that the C-terminal domain of PARP-1 is required for PARP-1 binding at genes with H2Av bearing nucleosomes (33). Thus, our ZnI, ZnII and AD-WGR constructs may not occupy the TSS of upregulated genes due to the absence of the catalytic domain. Next, we examined PARP-1 binding motifs at the promoters of differentially expressed genes in *Parp^C03256^* mutant animals that were highly occupied PARP-1 (clusters I and II; **Figure 2A**). At upregulated genes in *Parp^C03256^* mutants, PARP-1 peaks were associated with the E-box element, Motif 1 and a “CGTAA” motif (**Figure 4B**). In contrast, at downregulated genes, PARP-1 peaks were associated with Adh transcription factor 1 (Adf1), GAF and “GTGT” motifs (**Figure 4C**). Thus, our data suggests that while PARP-1 domains are less enriched at the TSS of upregulated genes, they may target PARP-1 to the promoters of genes activated by PARP-1 by targeting PARP-1 to GAF motifs (**Figures 1F**; **4D**). Taken together, our findings indicate that the cooperative action of the ZnI, ZnII, and AD-WGR domains results in targeted PARP-1 binding to the gene bodies of metabolic genes, leading to PARP-1-mediated repression. Additionally, these domains ensure that PARP-1 occupies the transcription start site and activates developmental genes that do not exhibit H2Av enrichment.

## Discussion

In this study, we aimed to investigate the involvement of specific PARP-1 domains and a ZnI mutant construct in the targeting of PARP-1 at steady-state in *Drosophila* third-instar larvae. Through our experiments, we determined that the ZnI domain is not essential for PARP-1 targeting to chromatin, and that the ZnII and AD-WGR domains are sufficient for targeting PARP-1 to chromatin except at active promoters. We also analyzed the binding patterns of PARP-1 to genes and found that it primarily binds to the promoters of active genes and the gene bodies of genes it represses. Additionally, our results indicate that the PARP-1 domains target PARP-1 to the gene bodies of active, lowly expressed, and upregulated genes, while targeting the TSS of downregulated genes. These findings suggest that the PARP-1 domains work together to regulate gene expression, with the ZnI, ZnII, and AD-WGR domains primarily targeting PARP-1 to the gene bodies of metabolic genes for repression and the TSS of developmental genes for activation.

The zinc fingers of PARP-1 differ from those of other zinc fingers in that they recognize the structure of DNA rather than a specific DNA sequence (1). The ZnI domain is a crucial component for PARP-1-mediated DNA activation. Interactions between the ZnI, ZnIII, WGR, and CAT domains are essential for PARP-1 activation in response to DNA damage. However, the ZnII domain is not essential for PARP-1-dependent DNA activation, but it has a significantly higher affinity for DNA (34). Thus, the ZnII domain may play a role in PARP-1 retention at sites of DNA damage (34) or targeting PARP-1 to chromatin in conjunction with other PARP-1 domains. This is consistent with our results that show the ZnI domain is not required for PARP-1 targeting to chromatin or PARP-1-regulated genes (**Figures 1B–E**, **4A**). Our previous studies have shown that the ZnI and ZnII domains in the DBD domain are necessary for full transcriptional activation mediated by PARP-1 at the *Hsp70* locus during heat shock (12). Therefore, while the ZnI domain may not be necessary for PARP-1 targeting, it may be crucial for PARP-1 activation during transcription.

The differential localization of PARP-1 domains to genes that are either repressed or activated by PARP-1 remains curious. PARP-1 is known to play a role in transcriptional regulation in various biological contexts, suggesting that it may have distinct binding modes for transcriptional activation and repression. Our previous research has shown that H2Av is necessary for the genome-wide localization of PARP-1 and that subsequent phosphorylation of H2Av activates PARP-1 (33). Additionally, the C-terminal but not the N-terminal DBD domain of PARP-1 is required to bind H2Av-bearing nucleosomes (12). PARP-1 binding was unaffected at the promoters of downregulated genes compared to upregulated genes. In contrast, PARP-1 domains were not enriched at the TSS of upregulated genes, but they had a higher occupancy at the TSS of downregulated genes. It is possible that ZnI, ZnII and AD-WGR are sufficient for binding to the gene bodies of H2Av-bearing genes that are repressed by PARP-1, while CAT is required for binding to their TSS. It has been demonstrated that the DBD and CAT cooperate to mediate chromatin compaction and repression *in vitro*, although the exact mechanism was not demonstrated (34). It is likely that the ZnI domain plays a crucial role in PARP-1’s ability to repress genes, similar to its importance in PARP-1’s activation during transcription and DNA repair. The ZnI domain may be necessary for PARP-1 to repress genes but not required for PARP-1 targeting to repressed loci. Thus, ZnI and ZnII may work together in a coordinated manner with other PARP-1 domains to target and activate PARP-1. Hence, ZnII may target PARP-1 to chromatin while ZnI activates PARP-1, creating a “one-two punch” effect.

## Conclusion

The study demonstrates the synergistic action of PARP-1 domains in targeting PARP-1 for the regulation of metabolic and developmental genes in third-instar larvae prior to pupation. Furthermore, it sheds light on the molecular mechanisms by which PARP-1 domains contribute to the chromatin-dependent functions of PARP-1.

## Data availability statement

The datasets presented in this study can be found in online repositories. The names of the repository/repositories and accession number(s) can be found below: GSE217730, GSE222877, GSE15292, GSE113278, GSE48510, GSE49488, GSE47260, GSE47258, GSE47259 (GEO).

## Author contributions

GB, SJ and AT conceived and initiated the project. GB and SJ performed the experiments. GB analyzed the data. GB and AT wrote the manuscript. GB and AT reviewed and edited the manuscript. GB and AT supervised the project. All authors contributed to the article and approved the submitted version.
